# A protocol for the measurement of myocardial blood volume and water exchange

**DOI:** 10.1186/1532-429X-13-S1-P140

**Published:** 2011-02-02

**Authors:** Octavia Biris, Neil Chatterjee, James Carr

**Affiliations:** 1 Northwestern University, Chicago, IL, USA

## Objective

Absolute myocardial perfusion MR imaging (ml/min/100g tissue) has the potential to timely diagnose and reduce patient mortality from coronary artery disease.

## Background

Organ perfusion can be quantified by direct calibration of relative perfusion images using absolute blood volume (ml/100g) [1]. It is well known that for an intravascular gadolinium-based T1 shortening contrast agent, the parenchymal T1 change reflects tissue blood volume [2].However, to accurately quantify blood flow from blood volume, we must describe the compartmentalization effects of intra- to extra-vascular water exchange [2,3].

## Materials and methods

### Protocol

In an instrumented dog we measured T1 using a cardiac gated Modified Look Locker Inversion Recovery (MOLLI)(4) pulse sequence (slice thickness 8 mm, FOV 171 x 343 mm2, matrix 96 x192, TR 173 ms, effective TI 100 ms ). Images were acquired on a 1.5 T Espree scanner (Siemens Medical Systems, Erlangen, Germany), during a short breath-hold, 5 minutes after injections of 0.003 mmol/kg of MS-325 (Ablavar, Lantheus Medical Imaging, Billerica, MA).

### Image processing

We estimated the myocardium and left ventricle blood pool T1 through fitting of MOLLI signal to the regrowth curves of the Look-Locker equation by an automatic image processing program developed in MATLAB R2009a (Mathworks, Natick, MA, USA).Myocardial blood volume (MBV) was calculated from the baseline to post-contrast change in T1 in the blood pool and myocardium.

## Results

**Figure 1:**
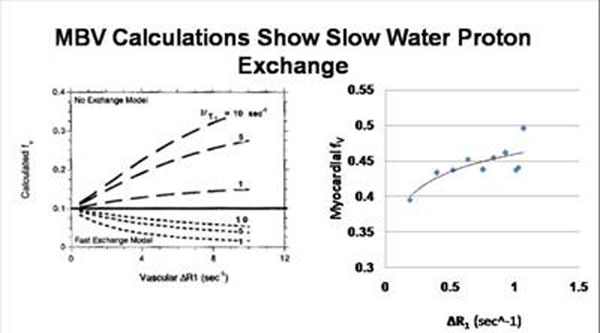
Vascular fractions fv predictions based on “No exchange” and fast exchange limits for a range of exchange values (left). Preliminary results from our experiments suggest the “No exchange” limit is appropriate for the quantification of myocardial blood volume (right).

Low dose injections (1/10th of single dose for humans) of MS-325 effected significant changes in myocardial T1’s (Table 1). The measured MBV was 40% of total myocardial volume, or 28 ml/100g, a value that over-estimates those quoted in the literature [5]. Water exchange in the myocardium was shown to approach the slow or no-exchange limit (Figure 1).

**Table 1:** Blood pool and myocardium T1

	Baseline	0.003 mmol/kg	0.009 mmol/kg	0.015 mmol/kg	0.021 mmol/kg	0.027 mmol/kg
T1 in left ventricle blood pool (ms)	1259±63	1018±33	761±15	645±19	580±16	548±33
T1 in myocardium (ms)	871±88	818±76	727±52	676±49	634±69	624±42

## Conclusions

We have established an imaging protocol to measure MBV and water exchange. Over-estimation of MBV may be caused by extravasation of MS-325, and to a lesser extent by T2 bias on the T1 measurements with the steady-state free precession MOLLI sequence. Future steps include measuring MBV with a strictly intravascular USPIO contrast agent, application of a more sophisticated fit that includes T2 effects, and determination of the water exchange constant by Monte-Carlo simulations.
